# A Rare Presentation of Benign Prostatic Hyperplasia With Pure Stromal Hyperplasia as a Large Isolated Pelvic Mass With Normal Prostate Imaging

**DOI:** 10.1177/10668969251407311

**Published:** 2026-01-13

**Authors:** Dong Ren, Roozbeh Houshyar, Robert A. Edwards

**Affiliations:** 1 Department of Pathology and Laboratory Medicine, 8788 University of California Irvine, Orange, CA, USA; 2 Department of Radiology, 8788 University of California Irvine, Orange, CA, USA

**Keywords:** benign prostatic hyperplasia, stromal hyperplasia, pelvic mass, prostate imaging, case report

## Abstract

Benign prostatic hyperplasia (BPH) is the most common benign lesion of the prostate in aging men. However, diagnosing BPH can be challenging when it presents as an exophytic pelvic mass, particularly if radiologic continuity with the prostate and prostatic glandular components are not clearly seen. Here, we report a 57-year-old male patient with a 2-year history of obstructive voiding dysfunction. Radiologic investigations, including both computed tomography and magnetic resonance imaging, revealed a large (up to 11.9 cm) solid pelvic mass located superior to the prostate with marked mass effect. No direct continuity with the prostate or multilocular/cystic structure was identified on imaging. Pathologic evaluation of the biopsy specimen showed a hypocellular lesion consisting of bland spindle cell proliferation and small blood vessels within a loose fibrous stroma. The subsequent resection specimen showed similar histologic morphology, and prostatic glands were not identified. Immunohistochemical staining demonstrated that the tumor cells were positive for CD34, desmin, androgen receptor, and progesterone receptor. Ki67 staining indicated a very low proliferative index (<1%). The constellation of histomorphological and immunohistochemical profiles was consistent with BPH with pure stromal proliferation. To the best of our knowledge, this represents the first documented instance of BPH with pure stromal hyperplasia manifesting as a distinct pelvic mass without radiologic continuity with the prostate. This case report highlights the need for clinicians and pathologists to consider BPH in the potential diagnosis of large pelvic lesions, especially in the condition that standard prostate imaging appears normal and prostatic glands are absent.

## Introduction

Benign prostatic hyperplasia (BPH), characterized by noncancerous nodular enlargement of the prostatic gland and stroma, is the most common benign lesion in men.^1^ It is almost ubiquitous in aging men, with an estimated prevalence of 60% by age 60% and 80% by age 80, as determined by autopsy evaluations.^1^ BPH typically presents with urinary symptoms either due to direct obstruction of the prostatic urethra—such as difficulty urinating, frequency, and urgency—or due to secondary changes in the bladder and/or kidneys resulting from chronically elevated intravesical pressure and hydronephrosis, leading to azotemia and renal failure.^1^ The transition zone, the innermost region of the prostate surrounding the urethra, is the most common site for BPH, as opposed to the peripheral zone, which is the site where most prostate cancer originate.^2^ As a result, the majority of BPH lesions present as primary prostatic nodules due to their central location within the prostate. However, lesions involving the peripheral zone^3^ and even extra-prostatic manifestations in the form of exophytic nodules,^4–6^ have been reported. Notably, all previous reports of BPH presenting as a pelvic mass have shown radiologic continuity with the prostate and prostatic glands on the histopathological examination. This case report is to describe an unusual presentation of BPH with pure stromal hyperplasia as a large, isolated pelvic mass without radiologic continuity with the prostate and lacking prostatic glands, which highlights the importance of considering a broad differential diagnosis, especially when conventional imaging suggests normal prostate morphology.

## Case Report

**Figure 1.**
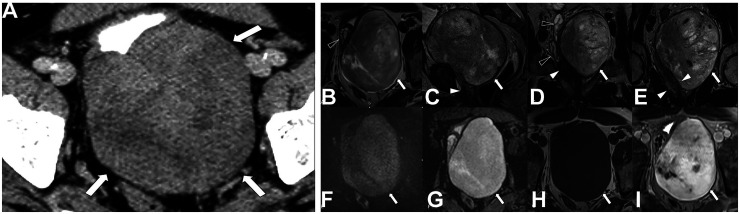
Radiologic evaluation of the pelvic mass. (A) Axial post contrast computed tomography shows a large heterogeneously enhancing mass (white arrows) superior to the prostate and bladder. (B-I) Magnetic resonance imaging of the pelvis with and without contrast (B—axial T2, C—sagittal T2, D and E—coronal T2, F—axial diffusion—weighted imaging, G—apparent diffusion coefficient, H—axial T1, I—axial T1 post contrast) demonstrates a large heterogenous T2 (B-E) intermediate mass (white arrows) superior to the left of the prostate (white arrowheads) with marked mass effect on the prostate and the seminal vesicles (black arrowheads), which are deviated anteriorly to the right. This mass demonstrates no restricted diffusion (F and G), is T1 hypointense (H) with heterogeneous enhancement (I).

A 57-year-old male patient presented with a 2-year history of obstructive voiding dysfunction, which acutely worsened approximately 3 months prior. A digital rectal exam revealed a large anterior mass without rectal invasion, and the prostate gland could not be palpated. Cystoscopy showed a normal-appearing bladder, but a small bulge was noted on the left side of the bladder. Computed tomography of the abdomen revealed a large, heterogeneously enhancing solid pelvic mass measuring up to 11.9 cm, located superior to the prostate and bladder and anterior to the rectosigmoid colon (Figure 1A). Subsequent magnetic resonance imaging of the pelvis confirmed the mass to be situated superior and to the left of the prostate, exerting significant mass effect on the prostate and seminal vesicles without evidence of continuity or invasion of the prostate (Figure 1B). No cysts or multilocular components were observed on imaging. The patient's prostate-specific antigen (PSA) level was 0.3 ng/mL, within the normal range (0-4.0 ng/mL).

The patient reported frequency and nocturia (2-3 times per night) but denied straining, sensation of incomplete bladder emptying, hematuria, abdominal pain, pelvic pain, back pain, fevers, chills, fatigue, weight loss, or night sweats. His past medical history was significant for hypertension, hyperlipidemia, a spinal fracture, and a traumatic left renal laceration. He denied smoking or alcohol use. Other laboratory values and family history were unremarkable.

**Figure 2.**
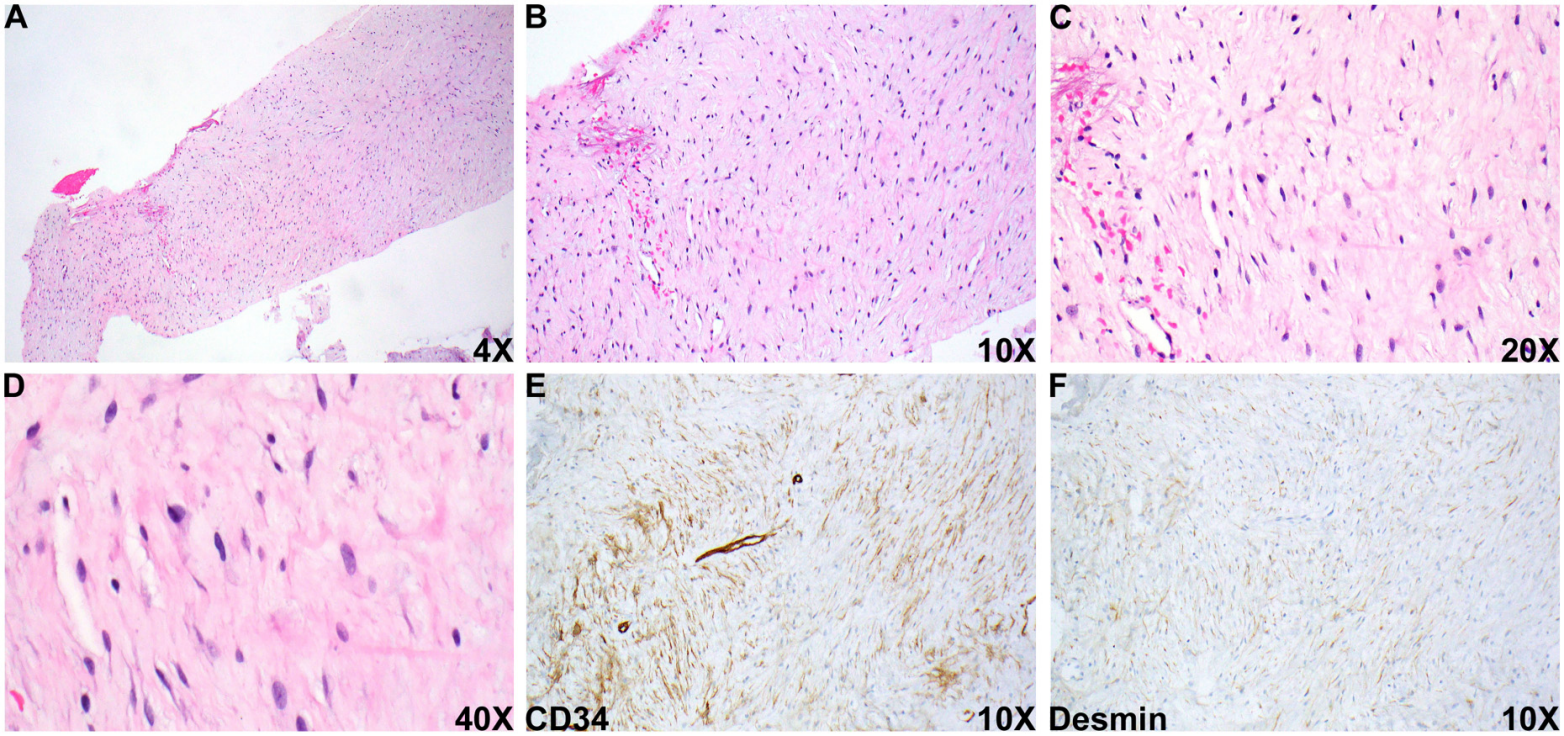
Histologic and immunohistochemical evaluation of the biopsy specimen. (A-D) Representative section of the biopsied lesion on H&E staining, 4 × (A), 10 × (B), 20 × (C), and 40 × (D). (E and F) representative section of immunohistochemical staining of CD34 (E) and desmin (F).

Pathologic evaluation of the biopsy specimen revealed a very hypocellular lesion, composed of bland spindle cell proliferation within a loose fibrous stroma (Figure 2A to D). Small arterioles and venules were present within the lesion. Extensive immunohistochemical staining showed the tumor cells were positive for CD34 and desmin (Figure 2E and F), but negative for SMA, S100, SOX10, HMB-45, EMA, GLUT1, KIT, STAT6, β-catenin, MUC4, and ALK (D5F3 clone). Ki67 staining indicated a very low proliferative index (<1%). Fluorescence in situ hybridization testing was negative for *MDM2* amplification.

**Figure 3.**
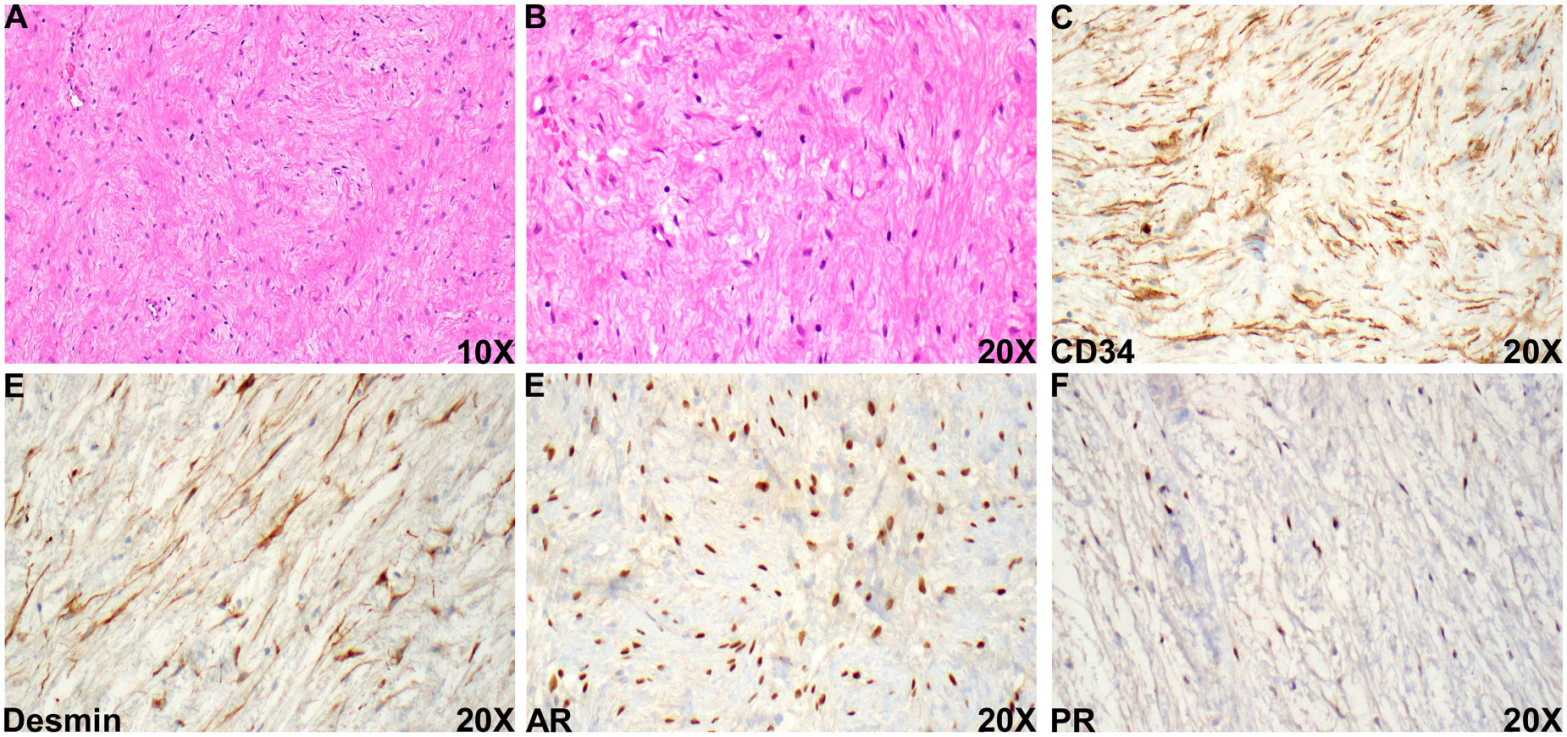
Histologic evaluation of the resection specimen. (A and B) Representative section of the resection specimen, 10 × (A) and 20 × (B). (C-F) Representative section of immunohistochemical staining of CD34 (C), desmin (D), androgen receptor (E), and progesterone receptor (F).

A resection specimen obtained 2 months later showed a homogenous lesion composed of bland spindle cell proliferation throughout the lesion within a loose fibrous stroma (Figure 3A and B). Prostatic glandular components, cytologic atypia, necrosis, and mitotic figures were not identified on the resection specimen. Immunohistochemical staining workup showed that the tumor cells were positive for CD34, desmin, androgen receptor, and progesterone receptor (Figure 3C to F), confirming its prostatic origin. PSA and PSAP failed to identify the prostatic glandular component, and the stromal cells were negative for SMA, S100, SOX10, β-catenin, MUC4, and ERG. RB1 was retained in the tumor cells. Ki67 staining was <1%. The morphological and immunohistochemical profiles favored the diagnosis of BPH with pure stromal hyperplasia. The specimen was reviewed by an outside institution for a second opinion, which concurred with the above diagnosis.

The patient had an uneventful postoperative course and remained asymptomatic at one-year follow-up after surgery.

## Discussion

Clinically, pelvic lesions encompass a wide range of conditions, from benign to malignant tumors and neoplasms, arising from the surrounding structures in the lower abdomen, including the gastrointestinal tract, genitourinary tract, gynecological organs (in female patients), and primary mesenchymal neoplasms of the pelvic area. Additionally, not all pelvic masses produce symptoms, and some may present with nonspecific symptoms, such as nausea, vomiting, abdominal bloating, swelling, pelvic pain, and changes in bowel or urinary habits. The differential diagnosis can vary based on gender and the duration of symptoms. Radiologic investigations play a crucial role in assessing the correlation between pelvic lesions and neighboring organs, aiding in determining the lesion's origin.^7^ However, the absence of abnormal radiologic findings does not exclude the possibility of a specific organ as the source. In this case report, both computed tomography and magnetic resonance imaging scans revealed a large, separate pelvic mass with no continuity with the prostate or any other organ. Histopathologic investigation confirmed the prostatic origin of the lesion, highlighting the importance of correlating radiologic and pathologic findings in clinical practice, especially when imaging results are inconclusive.

Tumor size plays a pivotal role in assessing pelvic masses.^8^ Although no definitive size cutoff exists to distinguish benign from malignant tumors, a mass larger than 10 cm often raises concern for malignancy, prompting differential diagnoses that include well-differentiated liposarcoma, dedifferentiated liposarcoma, leiomyosarcoma, and gastrointestinal stromal tumor. However, benign tumors, such as leiomyomas,^9^ solitary fibrous tumors,^10^ schwannoma,^11^ and hemangioma,^12^ are also reported as large pelvic masses over 10 cm in size. In this case report, we presented a large isolated pelvic mass measuring 11.9 cm on imaging, without clear continuity with surrounding structures. Pathologic evaluation of the biopsy revealed hypocellular proliferation of bland spindle cells that were positive for CD34, desmin, androgen receptor, and progesterone receptor, confirming the prostatic origin. Notably, giant multilocular prostatic cystadenoma presenting as large pelvic masses^13^ have been reported more frequently than BPH.^4–6^ However, the absence of a multilocular appearance on both imaging and pathology argues against this diagnosis, making BPH with pure stromal hyperplasia presenting as a large pelvic mass the most appropriate diagnosis in this case report.

Histologically, BPH typically appears as an encapsulated, well-demarcated multinodular lesion characterized by varying proportions of epithelial and stromal cell proliferation in the prostate gland.^3^ These nonspecific histomorphological features prompt a wide range of differential considerations, including, but not limited to, prostatic stromal tumor of uncertain malignant potential,^14^ giant multilocular prostatic cystadenoma,^15^ leiomyoma,^16^ and solitary fibrous tumor^17^ of the prostate. Notably, an equivocal case report of extraprostatic prostatic stromal tumor of uncertain malignant potential presenting as a large pelvic mass measuring 12 × 8 cm, extending rectally and suprapubically, without macroscopic connection to the prostate or microscopic evidence of prostatic tissue, has been reported.^18^ In this case report, pathologic evaluation of both biopsy and resection specimens revealed a purely bland, paucicellular spindle cell proliferation with prominent stromal capillary proliferation throughout the lesion, embedded within loose fibrous stroma- features suggestive of a benign process. Immunohistochemical staining workup demonstrated positivity for CD34, desmin, androgen receptor, and progesterone receptor, confirming the prostatic origin of the mass. Negative staining for SMA, STAT6, KIT, β-catenin, ALK, and MUC4 excluded leiomyoma, solitary fibrous tumor, gastrointestinal stromal tumor, desmoid fibromatosis, inflammatory myofibroblastic tumor, and low-grade fibromyxoid sarcoma of the prostate. Given these findings, the primary differential diagnosis included prostatic stromal tumor of uncertain malignant potential and BPH. Histologically, prostatic stromal tumor of uncertain malignant potential is characterized by a heterogeneous mixture of histologic patterns, including degenerative atypia, hypercellular stroma, myxoid changes, phyllodes-like architecture, and round cell morphology. Classically, atypical stromal cells in prostatic stromal tumor of uncertain malignant potential proliferate between benign prostatic glands—a feature not identified in this case report. Notably, prostatic glands may be absent in certain subtypes of prostatic stromal tumor of uncertain malignant potential, such as those with myxoid and stromal overgrowth patterns.^19–22^ In contrast, the presence of a multinodular growth pattern and prominent stromal capillary proliferation supports a diagnosis of BPH over prostatic stromal tumor of uncertain malignant potential. It is noteworthy that multilobated architecture can occasionally be seen in prostatic stromal tumor of uncertain malignant potential, and whether the multinodular features observed in biphasic epithelial and stromal BPH are also common in BPH with pure stromal hyperplasia remains unclear. Immunohistochemically, both BPH and prostatic stromal tumor of uncertain malignant potential may show variable expression of CD34, desmin, androgen receptor, and progesterone receptor.^23,24^ However, SMA positivity is observed in the majority of prostatic stromal tumors of uncertain malignant potential lesions (∼80%),^23^ and the Ki-67 labeling index typically ranges from 1% to 40% (mean 6%).^23,24^ In the present case report, SMA was negative, and the Ki-67 index was <1%. Taken together, the radiologic, histomorphologic, and immunohistochemical findings favor the diagnosis of BPH with pure stromal hyperplasia presenting as a large pelvic mass.

The lack of radiologic continuity with the prostate and the absence of prostatic glands within the lesion make the diagnosis of BPH particularly challenging in the context of a large pelvic mass. To our knowledge, all previously reported lesions of BPH presenting as exophytic pelvic nodules have demonstrated radiologic continuity with the prostate and the presence of prostatic glands, providing important clues to the prostatic origin.^4–6^ We hope this case report will raise awareness among pathologists and clinicians to consider BPH in the differential diagnosis of large pelvic lesions.

To our knowledge, this is the first reported case report of BPH with pure stromal hyperplasia presenting as a large, separate exophytic pelvic mass without radiologic continuity with the prostate. This case report underscores the importance of considering BPH as a potential diagnosis in solitary pelvic lesions, enhancing clinician and pathologist awareness.
